# When Disinfection Fails: Biocide Tolerance as a Driver of *Campylobacter* Persistence and Resistance

**DOI:** 10.3390/antibiotics15040357

**Published:** 2026-03-30

**Authors:** Inês M. Fonseca, Inês Martins, Mónica Oleastro, Susana Ferreira

**Affiliations:** 1 RISE-Health, Department of Medical Sciences, Faculty of Health Sciences, University of Beira Interior, Av. Infante D. Henrique, 6200-506 Covilhã, Portugal; ines.fonseca@ubi.pt (I.M.F.); ines.margarida.martins@ubi.pt (I.M.); 2 National Reference Laboratory for Gastrointestinal Infections, Department of Infectious Diseases, National Institute of Health Dr. Ricardo Jorge, Av. Padre Cruz, 1649-016 Lisbon, Portugal; monica.oleastro@insa.min-saude.pt

**Keywords:** *Campylobacter*, biocides, susceptibility, antimicrobial resistance, adaptive responses

## Abstract

*Campylobacter* spp. constitutes a significant global public health hazard as it is a leading cause of reported foodborne diseases. Human infection is predominantly acquired through the ingestion of contaminated food, unpasteurized milk and untreated water, prompting the widespread implementation of chemical disinfection across several sectors, from healthcare, domestic environments, and food-processing to animal husbandry. While these biocidal agents encompass multiples classes with different modes of action and efficacy, growing evidence suggests that their extensive and repeated use may unintentionally promote bacterial persistence, tolerance and adaptive responses. Although biocide resistance has been documented in several foodborne pathogens, data on biocide tolerance in *Campylobacter* spp. remain limited. Available studies report variable degrees of reduced susceptibility to commonly used biocides among isolates originating from poultry production, food-processing environments, and water systems. Importantly, while biocide-induced adaptive responses in *Campylobacter* spp. may potentially overlap with antimicrobial resistance mechanisms, the extent to which these agents drive co-selection, persistence, or dissemination requires further elucidation. Evidence remains limited on the effects of long-term and repeated exposure under realistic processing conditions, the interplay between stress-induced gene regulation and stable genetic changes, and the contribution of mobile genetic elements, biofilm formation, and microbial communities in shaping antimicrobial resistance evolution. In light of the global health burden imposed by campylobacteriosis and the rising challenge of antimicrobial-resistant *Campylobacter*, this review brings together current evidence on the role of biocides in shaping bacterial survival, adaptation, and resistance mechanisms.

## 1. Introduction

Foodborne diseases are a pressing concern for the World Health Organization (WHO), with 600 million cases of foodborne diseases and 420,000 deaths being reported in 2010, with an estimated loss of 33 million years of healthy lives related with the consumption of unsafe food [1]. This burden falls disproportionately on young children, with those under five years of age accounting for about 40% of all cases and an estimated 125,000 deaths annually. Moreover, the increasing globalization of the food trade, longer and more complex supply chains, climate change, and international travel heighten the risk of food contamination, facilitating the transboundary spread of foodborne hazards and escalating concern worldwide [1,2].

Over the past two decades, *Campylobacter* has been reported as a leading etiological agent of bacterial gastroenteritis worldwide and is consistently ranked by the WHO among the top four causes of diarrheal diseases [3]. In the European Union in 2024, campylobacteriosis was the zoonosis most frequently associated with reported hospitalizations and deaths among confirmed human cases and foodborne outbreak cases [4].

Despite its fastidious growth requirements, *Campylobacter* is able to persist in food-processing environments, where intensive disinfection practices are routinely applied, raising significant concerns regarding potential food product contamination and consequent adverse impact on human health [5,6]. This pathogen persistently infiltrates and thrives in production and processing environments, particularly those associated with poultry sources [7].

To mitigate this risk, chemical disinfection represents a cornerstone of hygiene and biosecurity in food production systems and animal husbandry, as well as healthcare facilities and domestic environments [8]. A wide range of biocides are routinely and extensively used across interconnected human, animal, and environmental settings, and their application has intensified in recent decades, also in response to increased food safety demands [9]. These compounds differ in their modes of action and efficacy and are essential for reducing microbial loads and limiting pathogen transmission. However, growing evidence suggests that widespread biocide use, especially under suboptimal conditions, may unintentionally contribute to bacterial persistence and adaptation rather than achieve complete eradication [10,11,12]. Consequently, bacteria manifest adaptive behaviors, potentially culminating in genetic alterations or activation of stress-response pathways, thereby fostering the acquisition of tolerance or even resistance to these chemical agents and raising concerns about antimicrobial resistance (AMR) selection. Several reviews on biocide resistance, resistance mechanisms or impact of AMR emergence have been published in recent years [12,13,14,15,16]; however, the extent to which biocide exposure drives *Campylobacter* adaptation and resistance evolution, as well as the mechanisms underlying these responses, remains underexplored.

Given the global burden of campylobacteriosis and the increasing prevalence of antibiotic-resistant *Campylobacter* strains, understanding how biocide use influences *Campylobacter* survival, adaptation, and resistance evolution is critical. Although studies report variable susceptibility to commonly used biocides, significant knowledge gaps remain. It is unclear whether repeated or sublethal biocide exposure promotes stable tolerance, co-selection of AMR, and long-term persistence under realistic food chain conditions, or how regulatory adaptation, genetic changes, mobile genetic elements and microbial community interactions contribute to these processes. These uncertainties directly limit the ability to develop evidence-based disinfection strategies and risk-mitigation policies across the farm-to-fork continuum.

This review therefore synthesizes current knowledge on biocide use, susceptibility patterns, and adaptive responses in *Campylobacter* spp., with particular emphasis on the food chain. By integrating evidence on biocide classes, tolerance and resistance patterns, and underlying mechanisms, this work aims to highlight key knowledge gaps.

## 2. Public Health Relevance of *Campylobacter*

From a public health perspective, *Campylobacter jejuni* and *Campylobacter coli* species are the primary species associated with human campylobacteriosis, together accounting for more than 98% of reported cases worldwide [17]. Although most cases are self-limiting, campylobacteriosis can be a particularly serious infection, and even evolve into severe post-infection complications [18]. The symptoms of *Campylobacter*-induced gastroenteritis range from watery or bloody diarrhea to abdominal cramping, fever, myalgia, and headache, with some patients experiencing nausea and vomiting, lasting six days to up to two weeks [19,20,21]. However, extraintestinal conditions like abscesses, meningitis, and bacteraemia can also occur in immunocompromised, pregnant, and elderly patients [19,22]. *Campylobacter* species may further contribute to chronic inflammatory gastrointestinal and periodontal diseases, and to some extent to celiac disease, inflammation of gall bladder, and colorectal cancer [20,23,24,25,26]. Besides gastrointestinal disorders, *Campylobacter* has been pointed out as the causative agent of postinfectious autoimmune disorders, such as reactive arthritis and Guillain–Barré syndrome, both with significant impairment in quality of life [21,27,28]. In addition, the burden of the disease extends to economic impact, with healthcare costs, productivity losses, and food safety concerns representing major challenges [29].

Despite being a long-standing problem, the impact of the disease remains far from under control, highlighting the urgent need for effective mitigation strategies. As reported by the European Food Safety Authority and European Center for Disease Prevention and Control, 24% of the 168,396 reported cases required hospitalization, with the highest notification rate recorded in children under five years, rendering the disease hyperendemic in this age group [4]. In the United States, the burden is similarly substantial, with an estimated 1.5 million cases annually reported by the Center for Disease Control and Prevention [30]. The economic impact is also considerable, with annual costs reaching up to €2.4 billion in the European Union and approximately USD 11.3 billion in the United States [31,32]. Despite regional variability, largely reflecting differences in diagnostic capacity, data collection, reporting practices, surveillance programs and accessibility to natural reservoirs, campylobacteriosis incidence remains consistently high in both developed and developing countries, as it is also becoming endemic in African, Asian, and Middle Eastern countries [20,29,33,34,35,36].

As the most frequently reported foodborne zoonoses, *Campylobacter* is a commensal bacterium inhabiting the intestinal tract of domesticated animals, wild animals, and livestock. Therefore, *Campylobacter* infection in humans is typically acquired through the consumption of contaminated food and water, and unpasteurized milk [37,38]. In fact, the preparation, handling or consumption of contaminated raw or undercooked poultry represents the main risk factor for sporadic cases of campylobacteriosis in humans, with 60 to 80% of cases attributed to chicken reservoir and its by-products [7,21,39]. Additionally, *Campylobacter*-associated outbreaks were mainly associated with poultry products in the EU and to dairy products in the US [4,40]. Other risk factors for sporadic campylobacteriosis include direct contact with farm animals, travel-related exposure or environmental exposure [41].

Although *Campylobacter* has strict physiological requirements and is subjected to numerous food safety intervention measures, it still develops adaptive strategies that enhance survival outside the host. This apparent paradox underscores the critical need for robust biosecurity measures spanning the entire food chain, from primary production to final consumption [5]. In poultry production, flock colonization typically occurs on farms through contaminated equipment, personnel, or environmental sources, whereas during transport and slaughter, cross-contamination of carcasses can arise via contaminated crates and through leakage of intestinal contents, equipment, and processing water [42,43]. Within poultry facilities, *Campylobacter* is required to survive in an aerobic environment, scalding, chilling and freezing, and under usage of antimicrobials [44]. Despite being sensitive to high oxygen concentrations in the normal atmosphere, clinically relevant *Campylobacter* strains increasingly display aerotolerance and enhanced resistance to oxidative stress [6,45,46,47].

Across agricultural sectors and along the food chain, *Campylobacter* is frequently exposed to biocides employed for disinfection and hygiene. This reliance on biocides amplifies the risk of selecting tolerant subpopulations and may facilitate the emergence of *Campylobacter* biocide-tolerant or -resistant strains [48,49,50,51]. Of particular concern is the potential for such exposure to modulate stress-response pathways that may overlap with antibiotic resistance mechanisms. These adaptive responses are particularly concerning in *Campylobacter*, for which therapeutic options are already constrained by rising AMR [36,52,53]. In fact, as a highly adaptable pathogenic organism, *Campylobacter* has developed increasing resistance to numerous clinically relevant antibiotics. This has led to the emergence of multidrug-resistant strains globally, complicating the management of severe infections and amplifying the public health impact of resistant *Campylobacter* circulating along the food chain [54,55,56]. Moreover, the detection of biocide and metal resistance determinants on mobile genetic elements, often together with antibiotic resistance genes, has raised concerns, in other foodborne pathogens, that the use of biocides in the food industry may select for strains carrying resistance to multiple antimicrobials [57,58].

Therefore, within a One Health framework, the widespread use of biocides may represent an additional selective pressure contributing to the persistence and dissemination of resistance traits across animal, environmental, and human reservoirs. Understanding how biocide exposure intersects with AMR is thus a critical, yet frequently overlooked, component of the AMR landscape in *Campylobacter* and a key step toward improving effective control strategies.

## 3. Biocides: General Applications, Modes of Action, and Resistance Mechanisms

### 3.1. Biocides and Their Applications

The use of chemical biocides has been vital for human safety and health for centuries, historically serving essential purposes such as material preservation, water and food purification and injury treatment, remaining integral to public health and food safety. These compounds are widely applied during primary production, on food-processing surfaces, in slaughterhouses, and within water systems to reduce microbial contamination and ensure food safety. Notably, similar biocidal agents are also extensively applied in domestic and healthcare environments, as well as in the oil and gas industries and consumer product manufacturing, resulting in continuous environmental exposure beyond food-associated settings [9,59,60].

The term biocide refers to chemically active substances employed to suppress, inactivate or eliminate a diverse range of undesirable or harmful organisms, including microorganisms, across domestic, industrial and clinical settings [59]. For the purposes of this review, biocide is defined as any active chemical agent used to control the growth of or kill bacteria in many environments, with particular emphasis on their use for environmental and surface disinfection, namely in the food chain [60].

Under European Union legislation, biocides can be organized into four main categories: (i) disinfectants whose primary role is eradicating established organisms; (ii) preservatives, whose function is to extend the lifespan of materials by preventing deterioration; (iii) pest control agents, which are, however, designed to chemically target and poison larger pest species; and lastly (iv) “other biocidal products”, a category that serves as a residual classification for any compounds not fitting into the previous categories [61,62]. Thus, antimicrobial biocides are a distinct class of chemical agents employed in preservation, antisepsis, and disinfection, not to be confused with chemotherapeutic antibiotics. While some biocidal compounds may be suitable for multiple applications, the term disinfectant specifically denotes a biocidal product applied to inanimate surfaces, whereas antiseptic refers to a product intended for use on or in contact with living tissues [63].

The global biocide market was valued at USD 8.9 billion in 2023 and is expected to rise to USD 12.9 billion by 2032. The water treatment sector led the market in 2023, with approximately 45% of the total share, driven primarily by the extensive need to manage microbial proliferation in municipal and industrial water-based systems. Geographically, North America accounted for 30% of the biocide market, whereas the Asia–Pacific region generated the highest revenue share, due to rapid urbanization, industrialization and high demand across agriculture, water treatment and industrial sectors [64]. Contributing factors to this increased demand include a growing public awareness of infection risks and microbial contamination, coupled with the escalating problem of antibiotic resistance [9].

In clinical settings, the widespread use of biocides is standard practice, with multiple applications, and they are employed for diverse purposes, including skin antisepsis, surface and water disinfection, equipment sterilization and formulation preservation [59].

Beyond the healthcare environment, biocides are used across various other sectors to manage microbial contamination and dissemination. In animal husbandry, for instance, these compounds are applied to farm vehicles, structures, barns and equipment to limit disease transmission and the spread of infectious outbreaks originating from the farms [9,65]. The food industry also relies heavily on biocides, where they function as important preservatives and key agents for controlling microbial contamination during production and processing, thereby safeguarding the food chain. This is particularly relevant for foodborne pathogens, such as *Campylobacter*, which is frequently associated with poultry production and contamination during slaughtering and processing. Consequently, effective sanitation and disinfection practices are critical to prevent the persistence of *Campylobacter* on food-contact surfaces and within processing environments, thereby reducing the risk of cross-contamination throughout the food production chain [42,43].

In both the water and food sectors, quaternary ammonium compounds (QACs) are among the most widely used disinfectants, due to their low toxicity and broad-spectrum antimicrobial activity [9,12]. Moreover, biocides are widely used as preservatives and disinfectants within household products and consumer goods, including personal care items, cosmetics, textiles and cleaning products. Among these, triclosan, employed in numerous medical and personal care products, has been one of the most studied agents [66,67,68]. Biocides are incorporated into several common daily items, appearing in products such as mouthwashes, socks, soap and surgical scrubs [12].

### 3.2. Major Classes of Biocides and Representative Compounds

Biocides comprise a chemically active and diverse group of substances, which although regulatory frameworks typically classify them into four categories, their extensive chemical diversity has led to the division of numerous classes tailored to specific applications. Currently, more than 900 distinct biocidal chemistries are available in Europe alone, underscoring both their diversity and adaptability [63].

Alcohols remain among the most widely used biocides, particularly in healthcare, owing to their rapid and broad-spectrum activity. Commonly used alcohols such as ethanol, isopropanol, and n-propanol are effective against bacteria, fungi, and viruses when applied at concentrations typically ranging from 59 to 90% (*w*/*v*) [69,70,71]. However, their volatility, flammability and toxicity at these concentrations may raise safety concerns, prompting the development of combinations with other biocides and excipients to enhance their effectiveness [61,71,72].

Aldehydes are a class of highly reactive and toxic chemicals derived from alcohols, valued for their antimicrobial activity but constrained by significant toxicity [73,74]. The most employed aldehydes consist of glutaraldehyde and ortho-phthalaldehyde. Glutaraldehyde has been widely used for the disinfection of heat-sensitive and semi-critical medical equipment due to their effectiveness against bacteria, mycobacteria, viruses, fungi, and, in some cases, spores [73,75]. Ortho-phthalaldehyde has been proven to be more suitable and to eradicate a variety of microorganisms. When combined with benzyldimethyldodecylammonium chloride, it showed promising results as an effective, non-toxic disinfectant [75,76,77]. Despite their efficacy, concerns related to skin and respiratory sensitization have driven the search for safer formulations and alternative agents [73].

Several phenolic compounds, including anilides, bisphenols, halophenols and phenols, have historically played a central role in antimicrobial control. Triclocarban and triclosan, for example, have been extensively incorporated into personal care and household products due to their activity against non-sporulating bacteria and fungi [78,79,80], but also in food packaging materials, food industry floors or medical supplies [81,82]. However, the environmental persistence, bioaccumulation, and endocrine-disrupting potential of these compounds have raised substantial ecological and public health concerns, leading to regulatory restrictions [80,81,82,83,84]. Similarly, phenolic compounds, despite their historical significance dating back to early antiseptic practices, are now recognized as environmentally hazardous pollutants, particularly chlorophenols and nitrophenols, which are classified as priority contaminants by regulatory agencies [85,86].

Biguanides are a versatile and widely used class of biocides, being applied in a variety of broad-range pharmaceuticals, as well as antiseptics and disinfectants, due to their antibacterial activity [87]. Agents such as chlorhexidine, alexidine, and polyhexamethylene biguanide (a mixture that consists of polymeric biguanides with amine, cyanoguanine and guanidine groups) exhibit broad-spectrum antimicrobial activity and are commonly employed in wound care, oral hygiene, contact lens solutions, surface disinfection, and water treatment [87,88,89].

Halogen-releasing agents, particularly chlorine- and iodine-based compounds, are commonly applied as antiseptics, preservatives and disinfectants, especially in healthcare, but also in water treatment and the food chain [61,90,91]. Chlorine compounds are extensively used for surface disinfection and water sanitation, while iodine-based formulations, particularly povidone–iodine, are valued for their rapid and broad-spectrum antimicrobial effects [61,92]. Nevertheless, their efficacy can be compromised in the presence of organic matter, and improper use may lead to unintended by-products with environmental implications [92,93].

Chelating agents such as ethylenediaminetetraacetic acid are not recognized as potent antimicrobial compounds on their own, but they have a synergistic effect when combined with other biocides, common preservatives, antibiotics and cationic surfactants. Their ability to destabilize microbial membranes and biofilms has led to widespread use in contact lenses disinfection, wound care, and biofilm-associated conditions [94,95].

Heavy metal derivatives, including copper, silver, and mercury compounds, represent some of the oldest antimicrobial agents, exerting activity through oligodynamic effects, and antimicrobial activity depends on the oxidative state of the metal [61,96]. While these metals remain valuable in medical devices, wound care, and water systems, their persistence and toxicity pose significant ecological risks [61,96,97,98]. Importantly, environmental exposure to heavy metals can co-select for antimicrobial resistance genes, thereby indirectly contributing to the global antimicrobial resistance crisis [98].

Peroxigens are considered to be one of the most environmentally friendly class of biocides, since their complete breakdown results in water, which poses minimal risk to the environment [61]. Hydrogen peroxide and peracetic acid are the two most common peroxigens used throughout multiple sectors, being increasingly favored due to their broad-spectrum efficacy against viruses, fungi and bacteria [99]. These agents are widely used for antisepsis, disinfection and sterilization, mainly within the food industry, healthcare settings and water supplies [61,99]. Nonetheless, issues related to stability and corrosiveness require careful formulation and handling. Peracetic acid is a potent antimicrobial compound even at very low concentrations; however, it usually needs to be paired with either water, acetic acid or hydrogen peroxide to be used for disinfection purposes [61,99].

Lastly, QACs remain among the most widely used biocides across industrial, healthcare, and domestic settings [100]. Their antimicrobial activity is closely linked to molecular structure, particularly alkyl chain length, enabling modified applications against diverse microorganisms [100,101]. The most commonly used is benzalkonium chloride, which is one of the main constituents in disinfectants, both in clinical and agricultural environments, and is composed of a mixture of different alkyl chain lengths [61,102]. However, concerns regarding toxicity, environmental persistence, and the emergence of AMR have led to increasing analysis of their widespread use [102].

### 3.3. Biocidal Modes of Action

To be effective, biocides must reach and interact with microbial target sites. In contrast to what happens with antibiotics, biocides at their in-use concentration exhibit a broader spectrum of activity, through multi-target modes of action. Usually, these series of events initiate at the cell surface and progress through the cytoplasmic membrane to intracellular components, resulting in rapid and often irreversible cellular damage [16,103]. The cell envelope represents the first line of interaction between biocides and the cell and is frequently the primary site of inactivation [16]. Structural differences between Gram-positive and Gram-negative bacteria strongly influence susceptibility. Gram-negative bacteria exhibit a rigid and hydrophilic barrier to many biocides due to the presence of an outer membrane rich in lipopolysaccharide (LPS), stabilized by divalent cations, thus limiting the penetration of hydrophobic compounds [104,105]. Entry of biocides is therefore dependent on compound properties, such as molecular size, charge, and hydrophobicity, with small hydrophilic molecules diffusing through porins, while larger or lipophilic agents face restricted access [106]. Destabilization of the outer membrane enhances biocide uptake and can be facilitated by chelating agents such as ethylenediaminetetraacetic acid. This process is frequently dependent on multiple factors, including biocide concentration, temperature, formulation and pH, which can alter the ionizing state of the agent employed and the charge of the microbial cell, thereby influencing binding affinity [106].

Many commonly used biocides, such as aldehydes, biguanides and QACs, interact directly with outer membrane components altering its charge and hydrophobicity, displacing essential cationic components, resulting in the deformation of the cell wall and membrane, and consequently leakage of the intracellular components [61,103]. Biocides that are able to damage the outer cell membrane and cell wall promote their own uptake to reach target sites inside the cytoplasm [103]. The cytoplasmic membrane represents a vital physiological target for various biocides, such as phenols, biguanides, QACs and alcohols, as it is a semi-permeable barrier responsible for osmoregulation, active transport and maintenance of the proton motive force [104].

Cationic agents typically combine a strong positive charge with a hydrophobic domain to be able to penetrate the cytoplasmic membrane [87]. The loss of cytoplasmic membrane integrity is frequently reflected in the efflux of cellular contents, with K+ being released first, followed by inorganic phosphates, amino acids and nucleic acids [104]. At higher concentrations, membrane disruption facilitates penetration into the cytoplasm, where additional intracellular damage occurs, as observed with biguanides and phenolic compounds [63,103]. Alcohols primarily act by denaturing membrane-associated essential proteins and disrupting membrane and enzymatic functions [103]. Oxidizing agents, including chlorine, iodine and peroxygens, damage membranes indirectly through oxidation of membrane proteins and lipids, while simultaneously impairing enzymatic activity and nucleic acid synthesis [63]. A key consequence of membrane damage is dissipation of the proton motive force. Several biocides, including phenols, QACs, biguanides, alcohols, and weak organic acids, lead to energy depletion and metabolic failure, allowing for the cell to lose the ability to perform active transport, therefore preventing the uptake of nutrients and the expulsion of toxic compounds [63,103,104,107].

Once biocides pass the cell envelope, they may interact with the intracellular constituents. Alkylating and oxidizing agents are highly reactive, reacting with several groups of bacterial proteins and nucleic acids. For example, glutaraldehyde, ortho-phthalaldehyde and formaldehyde are compounds that will contribute to intermolecular cross-links with proteins and nucleic acids, whereas hydrogen peroxide forms hydroxyl radicals, oxidizing thiol groups in both proteins and enzymes. In addition to hydrogen peroxide, both halogens and peracetic acid interact with cellular components [104,108]. However, beyond enzymes and nucleic acids, there are multiple biocides that also interact with ribosomes, which, despite not being the primary target, are susceptible to biocides and therefore may be damaged by hydrogen peroxide, proflavine and ρ-chloromercuribenzoate [103]. Alcohols further contribute to intracellular damage through protein denaturation, a process enhanced in aqueous solutions [69]. Furthermore, ethanol may also interfere with metabolic pathways by inhibiting and uncoupling mRNA and protein synthesis through the impact it exerts on ribosomes and RNA polymerase [109].

### 3.4. Bacterial Resistance Mechanisms

Despite the broad and multi-target nature of biocidal activity, microorganisms can withstand biocidal exposure through both intrinsic and acquired resistance mechanisms [61,63]. Physical permeability barriers, enzymatic neutralization, biofilms and active efflux pumps are considered, mostly, innate defenses, whereas acquired mechanisms are gained through target alteration, including mutations that alter cellular components, or the uptake of resistance genes through horizontal gene transfer [110].

In vitro research on bacterial tolerance or resistance has been performed with microorganisms, such as *Pseudomonas aeruginosa*, *Proteus mirabilis*, *Staphylococcus aureus* and *Escherichia coli*, across a broad range of biocides, including phenolics, such as triclosan, QACs, glutaraldehyde, chlorhexidine and oxidizing agents [111,112,113,114,115,116]. This interaction is based on the physiological stress biocides cause on bacterial cells, which triggers a variety of protective mechanisms to prevent the detrimental effects [9].

Intrinsic resistance arises from structural features such as the LPS-rich outer membrane of Gram-negative bacteria, the mycolic acid layer of mycobacteria, and the thick peptidoglycan of Gram-positive bacteria, all of which limit penetration [117,118,119]. Sporulation further enables certain bacteria to withstand extreme chemical stress, posing significant challenges in healthcare and the food chain [120].

Adaptive responses to biocides include cell surface alterations and permeability modifications from Gram-positive and Gram-negative bacteria to mycobacteria, such as alterations in membrane composition or porin expression [117,121]. Changes in outer membrane proteins following exposure to biocides, such as benzalkonium chloride, mirror those observed during antibiotic adaptation, highlighting shared physiological stress responses [122,123].

Efflux pumps serve as specialized transport systems whose main function is to expel toxic substances, including biocides, from the bacterial cytoplasm, thus lowering their intracellular concentrations [61,63]. Multiple efflux pump families contribute to this process, with the resistance-nodulation-division family (RND) predominating in Gram-negative bacteria and Major Facilitator Superfamily (MFS) transporters in Gram-positive bacteria [124]. Examples of this adaptive response are the upregulation of efflux-related genes post-exposure to triclosan in *E. coli* and *Salmonella enterica* [125] or the potential role of the qac transporter, part of the Drug/Metabolite Transporter (DMT) family as it functions as an exporter for lipophilic cations, such as QACs [126].

Horizontal gene transfer further accelerates the dissemination of biocide tolerance and resistance genes across microbial communities, often in conjunction with antibiotic resistance determinants [127]. Plasmid-mediated transfer of genes encoding QAC efflux pumps alongside β-lactamases exemplifies the convergence of biocide and antibiotic resistance [124,128].

Finally, biofilm formation may provide a strong defense against biocidal action. The extracellular polymeric substance matrix functions as a physical barrier, restricting penetration, neutralizing reactive compounds, or supporting the persistence of tolerant subpopulations. Biocide efficacy within biofilms varies depending on chemical properties and interactions with the matrix, emphasizing that penetration alone does not fully predict antimicrobial performance [63,129,130].

## 4. Biocide Tolerance in *Campylobacter* spp.

In the context of global efforts to control pathogens, the extensive use of biocides across diverse settings has become a subject of debate, particularly regarding whether repeated and sublethal exposures may promote bacterial survival, adaptive resistance and potential development of resistance [10,11,12]. Although biocide resistance among different foodborne pathogenic bacteria has been vastly reported, data about biocide tolerance or resistance in *Campylobacter* spp. is limited. Varied degrees of tolerance or reduced susceptibility to commonly used biocides have been observed. This phenomenon has been reported across multiple biocide classes, particularly in isolates originating from poultry production, food-processing environments, and water systems. Substantial levels of reduced susceptibility among *Campylobacter* isolates after cleaning and disinfection procedures at all stages of the food chain have been recorded [43,131].

### 4.1. Challenges and Knowledge Gaps in Campylobacter Biocide Susceptibility Assessment

Despite increasing research on the susceptibility of *Campylobacter* to biocides, the available evidence remains limited and fragmented. Most studies address isolated aspects, such as susceptibility profiling, selected molecular mechanisms, or phenotypic outcomes, rather than providing an integrated assessment of biocide tolerance in this pathogen.

Cross-study comparisons are further hindered by substantial methodological heterogeneity. Variations in biocide formulations, as well as differences in exposure conditions, such as concentration, contact time, temperature, organic load and application format, can markedly influence the measured antimicrobial activity of the biocides and therefore limit the direct comparability of experimental results and complicate the interpretation of reported susceptibility patterns. Although internationally standardized methods exist to evaluate the antimicrobial efficacy of biocides prior to market authorization, such as EN1040 and EN1276 quantitative suspension tests and the EN 13697 for quantitative surface tests, these protocols are primarily designed to assess product performance under defined conditions rather than to establish susceptibility thresholds. Moreover, these frameworks rely on a panel of reference microorganisms and do not include *Campylobacter* spp. as a test organism. To date, these standardized frameworks have not been routinely used to evaluate the activity of biocides against *Campylobacter* [132,133,134,135].

Another fundamental challenge in assessing global patterns of biocide tolerance in *Campylobacter* lies in the definition of “resistance” itself. Unlike antimicrobial resistance to antibiotics, where resistance is defined by clinically relevant breakpoints, biocide resistance is described using a range of overlapping terms, including “resistance”, “tolerance”, “reduced or decreased susceptibility”, “insusceptibility”, “non-susceptibility”, and “acquired reduced susceptibility”. This lack of terminological consensus reflects methodological variability and contributes to difficulties in the understanding of bacterial resistance to biocides [9]. In practice, however, the survival of bacteria following exposure to a biocidal product, particularly under real-world conditions, may be interpreted as functional resistance, regardless of the terminology used [11,136,137].

In many cases, biocide resistance has been inferred from modest shifts in minimum inhibitory or bactericidal concentrations (MIC or MBC), even when biocides remain effective at their in-use concentrations [138,139]. Tolerant or resistant phenotypes are generally inferred from comparative MIC analyses, often relying on reference strains or data derived from other bacterial species [132,133,134,135]. This complicates the differentiation between natural variability in susceptibility, but also in true adaptive responses to biocide exposure.

Thus, given the current limitations in biocide susceptibility assessment, including the lack of standardized testing protocols, the frequent evaluation of pure substances despite most of the commercial biocides being complex formulations, and the absence of consistent terminology, clear operational definitions are required. Accordingly, this review defines biocide “resistance” as the failure of an in-use biocide concentration to inhibit or kill a bacterial isolate, and, in contrast, “tolerance” as a broader descriptive term referring to the ability of bacteria to survive transient exposure to a biocide, often at higher concentrations, without necessarily stable shifts in susceptibility parameters [140].

This lack of organism-specific benchmarking remains a key knowledge gap, limiting the accurate assessment of the prevalence and public health significance of biocide tolerance in this foodborne pathogen [141]. Nevertheless, the observed patterns underscore the complexity of *Campylobacter* biocide tolerance and its potential relevance for persistence in food production environments (Table 1).

### 4.2. Campylobacter spp. Susceptibility to Biocidal Activity

In studies assessing susceptibility profiles of poultry-isolated *C. jejuni* and *C. coli* isolates, almost all strains showed non-susceptibility to triclosan at concentrations higher than 4 µg/mL, 32 or 96% of strains were non-susceptible to chlorhexidine at concentrations equal to or higher than 1 µg/mL, and all the strains were susceptible to benzalkonium chloride at concentrations below 30 µg/mL [132,133]. It is important to note that, in these studies, “susceptibility” was defined relative to empirical MIC distributions and previously used cutoffs in related Gram-negative bacteria [132,133]. Similarly, in Avrain et al.’s (2003) study [142], whose aim was to adapt a filtration method for determining disinfectant susceptibility in *Campylobacter*, all the 34 strains of *C. jejuni* and *C. coli* tested were susceptible to 1% benzalkonium chloride and 0.63% sodium hypochlorite. In this study, “susceptibility” was assessed when the reduction in viable cells after five minutes of contact with disinfectants was more than 10^5^ cells [142]. In contrast, Zhou et al. (2018) [134] demonstrated that 15% of the strains were susceptible to benzalkonium chloride at concentrations >1 µg/mL, significantly higher than for cetrimonium bromide, for which 24% of strains were non-susceptible at concentrations higher than 32 µg/mL. In this case, *C. jejuni* and *C. coli* non-susceptible strains were identified by comparing MIC values to those of positive control *C. jejuni* reference NCTC 11168, assessed by the agar dilution method. This was complemented with the detection of genes associated with biocide resistance in *Campylobacter* and other food-related bacteria, such as *S. enterica* and *E. coli* [134].

Notably, regardless of the susceptibility rate, Beier et al. (2019) [132] observed a clear trend toward increased biocide tolerance over time, as evidenced by a progressive tendency to higher MIC_50_ and MIC_90_ values for several antimicrobial substances tested in *C. coli* isolates from swine and commercial pork chops collected between 1998 and 1999 and 2015 (Table 1). This temporal rise in MIC_50_ and MIC_90_ values suggests a population-level shift in susceptibility, consistent with an increased prevalence of isolates displaying reduced biocide susceptibility and necessitating higher concentrations to inhibit growth compared with earlier isolates. Importantly, this trend was observed across multiple biocidal agents, including triclosan, benzalkonium chloride and chlorohexidine [132].

In addition to poultry strains, an investigation into the prevalence of resistance among *C. coli* and *C. jejuni* from food, animal, human, and environmental water sources reported heterogeneous susceptibility to disinfectants such as triclosan, benzalkonium chloride, chlorhexidine diacetate, trisodium phosphate, cetylpyridinium chloride, and sodium dodecyl sulphate. Although some differences between species were observed, no consistent association between antibiotic resistance and biocide tolerance was identified in that dataset [135]. In the study by Mavri et al. (2012) [135], biocide susceptibility was inferred from MIC distributions and comparative analyses with other bacterial organisms. Even with the variation in susceptible levels, all the tested strains proved to be non-susceptible to triclosan at concentrations higher than 8 µg/mL, while only one human clinical multi-resistant isolate was non-susceptible to benzalkonium chloride at a concentration of 4 µg/mL, aligning with those reported for *S. enterica* and *Listeria monocytogenes* [143,144,145,146,147]. In contrast, the MIC values for the biocides tested were generally lower than those reported for *Acinetobacter*, *Citrobacter* and *Pseudomonas* industrial isolates, *Pseudomonas stutzeri*, and *Campylobacter* isolates from slaughterhouses [43,147,148]. While previous reports noted MIC reaching 100–340 µg/mL for several biocides, this study revealed MIC ranges between 0.016 and 4 µg/mL for benzalkonium chloride, cetylpyridinium chloride, and chlorhexidine diacetate, whereas elevated triclosan MICs of 8–64 µg/mL endured. These findings underscore species-specific differences in biocide susceptibility and highlight the challenges associated with cross-study and cross-species comparisons [135]. With respect to the species, *C. coli* strains were in general more resistant than *C. jejuni* strains. Furthermore, biocide resistance did not significantly differ according to antibiotic resistance profiles, except for sodium dodecyl sulphate, for which higher tolerance was observed in erythromycin-sensitive and non-multidrug-resistant strains compared with resistant ones [135].

Importantly, the MIC values reported in experimental studies should be interpreted in the context of in-use biocide concentrations applied in the food chain. For *Campylobacter*, systematic comparisons between laboratory susceptibility thresholds and manufacturer-recommended industrial concentrations remain scarce, limiting assessment of the practical relevance of the observed MIC distributions. Evidence from other foodborne pathogens provides a useful perspective. For instance, recent work on *Listeria monocytogenes* demonstrated that recommended in-use concentrations of many biocides substantially exceed MIC values, suggesting that tolerance selection is unlikely under properly implemented sanitation regimes, whereas sublethal exposure resulting from inadequate cleaning or improper dosing may represent a more realistic risk scenario [149]. Similar integrated evaluations are largely lacking for *Campylobacter*, representing an important knowledge gap for risk assessment in food production settings.

### 4.3. Biocidal Intervention in Poultry Processing

In some countries, chemical antimicrobial interventions are applied for carcass decontamination during poultry processing, with peracetic acid being one of the most effective agents used to reduce contamination by pathogens such as *Campylobacter* [150]. Although this biocide completely inhibits the in vitro growth of *C. jejuni* and *C. coli* at concentrations as low as 8 ppm, substantially higher concentrations are required under industrially relevant conditions to achieve a meaningful reduction in this pathogen on chicken breast and drumstick skin. In some cases, concentrations up to 150-fold higher are needed, particularly when factors such as temperature and modified-atmosphere packaging are considered [151]. At these higher concentrations (400 and 1200 ppm), peracetic acid has been shown to effectively reduce pathogen loads on ground chicken and in post-chill water without negatively affecting the sensory quality of the product [152,153,154]. In contrast, chlorine, although widely used as a standard biocide in the poultry industry, shows more variable and often incomplete efficacy [8,152]. According to recent in vitro research, chlorine may successfully reduce *C. jejuni* in chicken carcasses prior to washing, but notably without achieving a “zero” infection level at concentrations between 0.2 and 50 ppm. Its effectiveness is strongly influenced by bacterial load, genetic variability, and strain-specific tolerance mechanisms [8,152,155].

Despite measurable reductions in viable *Campylobacter* counts following treatment with commonly used biocides, including sodium hypochlorite, peracetic acid, and acidified sodium chlorite, complete pathogen elimination is frequently not achieved. In fact, although biocide exposure at 40 or 100 ppm for 2 or 15 min decreases cultivability, the total number of *Campylobacter* cells, considering both live and dead cells, attached to chicken skin can remain largely unchanged, indicating persistence of bacterial biomass and the potential presence of non-culturable cells [156]. Similarly, carcass washer systems employing multiple sequential washes have been shown to provide only limited reductions in *Campylobacter* populations during poultry processing. While more aggressive interventions, such as acidified sodium chlorite applied at 1200 ppm or 12% trisodium phosphate solution with a contact time of 15 s, result in significantly greater reductions, these approaches still fail to fully eradicate *Campylobacter* from raw poultry [157]. Collectively, these findings underscore the inherent limitations of current chemical decontamination strategies and highlight the continued risk of *Campylobacter* persistence on poultry products despite intensive biosecurity measures.

**Table 1:** Overview of reported biocide tolerance in *Campylobacter* spp. summarizing proposed resistance mechanisms.

Biocide Class or Chemical Category	Compound	Tolerance or Resistance Rate	MIC Range/MIC_50_/MIC_90_	Mechanisms of Tolerance or Resistance	References
Halophenols	Triclosan	Almost all poultry strains non-susceptible at concentrations > 2 µg/mL ^a^	MIC range: 0.5–64 µg/mL, MIC_50_: 32 µg/mL and MIC_90_: 32 µg/mL	-	[133]
		MIC_50_ and MIC_90_ increasing over time	MIC range: 16–>64 µg/mL, MIC_50_: 32 and MIC_90_: 64 µg/mL (1998–1999); MIC range: 16–>64 µg/mL, MIC_50_: 64 µg/mL and MIC_90_: 64 µg/mL (2015)		[132]
		All food, animal, human and environmental water strains non-susceptible at concentrations > 8 µg/mL; MIC values slightly lower than those reported for other bacterial species ^b^	MIC range: 8–64 µg/mL		[135]
Biguanides	Chlorohexidine	32% of poultry strains non-susceptible at concentrations ≥ 1 µg/mL ^a^	MIC range: 0.25–8 µg/mL, MIC_50_: 0.5 µg/mL and MIC_90_: 1 µg/mL	-	[133]
		84% (1998–1999) and 96% (2015) of poultry strains non-susceptible at concentrations ≥ 1 µg/mL ^a^ MIC_50_ and MIC_90_ increasing over time	MIC range: 0.12–4 µg/mL, MIC_50_: 1 µg/mL and MIC_90_: 2 µg/mL (1998–1999); MIC range: 0.5–8 µg/mL, MIC_50_: 2 µg/mL and MIC_90_: 4 µg/mL (2015)		[132]
		MIC values lower than those reported for other bacterial species ^b^	MIC range: 0.063–2 µg/mL		[135]
		Tolerance observed in biofilms at a concentration of 10,000 µg/mL ^¶^ for 13% of the poultry strains tested	^†^	Biofilm-mediated protection	[50]
QACs	Benzalkonium chloride	All poultry strains susceptible at concentrations < 30 µg/mL ^a^	MIC range: ≤0.25–8 µg/mL, MIC_50_: 1 µg/mL and MIC_90_: 2 µg/mL		[133]
		All poultry strains susceptible at concentrations < 30 µg/mL ^a^ MIC_50_ and MIC_90_ increasing over time	MIC range: ≤0.25–16 µg/mL, MIC_50_: 1 µg/mL and MIC_90_: 4 µg/mL (1998–1999); MIC range: ≤0.25–16 µg/mL, MIC_50_: 2 µg/mL and MIC_90_: 8 µg/mL (2015)		[132]
		All poultry strains susceptible at 10,000 µg/mL ^c,¶^	^†^		[142]
		85% of poultry strains non-susceptible at concentrations > 1 µg/mL ^d^	MIC range: 0.25–64 µg/mL ^#,¶^	No consistent link to the presence of genes encoding resistance to quaternary ammonium compounds (*qac* genes)	[134]
		98% of food, animal, human and environmental water strains susceptible at ≤2 µg/mL; MIC values lower than those reported for other bacterial species	MIC range: 0.016–4 µg/mL		[135]
		Incompletely inhibition or eradication of biofilms (concentration of 100,000 µg/mL % ^¶^ after 10 min of exposure across 1–25 °C), with surface water less susceptible than slaughterhouse-derived isolates	^†^	Biofilm-associated cells exhibited lower efflux pump activity compared with planktonic cells Biofilm protection	[158,159]
	Cetylpyridinium chloride	MIC values lower than those reported for other bacterial species ^b^	MIC range: 0.25–4 µg/mL	-	[135]
	Cetylpyridinium bromide	All poultry strains susceptible ^a^	MIC range: 0.5–16 µg/mL, MIC_50_: 4 µg/mL and MIC_90_: 8 µg/mL	-	[133]
		MIC_50_ and MIC_90_ increasing over time	MIC range: 0.5–8 µg/mL, MIC_50_: 2 µg/mL and MIC_90_: 8 µg/mL (1998–1999); MIC range: 0.5–16 µg/mL, MIC_50_: 4 µg/mL and MIC_90_: 8 µg/mL (2015)		[132]
	Cetrimonium bromide	24% of poultry strains non-susceptible at concentrations > 32 µg/mL ^d^	MIC range: 0.125–1024 µg/mL ^#,¶^	No consistent link to the presence of genes encoding resistance to quaternary ammonium compounds (*qac* genes)	[134]
Halogen-releasing agents	Sodium hypochlorite	All poultry strains susceptible at 6300 µg/mL ^c,¶^	^†^	-	[142]
		Reduction but no complete elimination of viable *Campylobacter* counts during poultry processing (40 or 100 µg/mL ^¶^ for 2 or 15 min) Complete killing at 2000–4000 µg/mL		-	[156,160]
		Tolerance observed in biofilms at a concentration of 10,000 µg/mL ^¶^ for 20% of the poultry strains tested		Biofilm-mediated protection	[50]
	Chlorine	Variable and often incomplete efficacy in poultry industry (0.2–50 µg/mL ^¶^), with recovery following exposure reported	^†^	-	[7,152,155]
		-		-	[161]
		Completely inactivated biofilms after treatment at 50 µg/mL ^¶^ for 45 s		-	[162]
	Acidified sodium chlorite	Reduction but no complete elimination of viable *Campylobacter* counts during poultry processing (40 or 100 µg/mL ^¶^ for 2 or 15 min, nor at 1200 µg/mL ^¶^ for 15 s)	^†^		[156,157]
		^-^		-	[161]
	Chlorous acid	Complete killing at 200–400 µg/mL ^¶^	^†^	-	[160]
Peroxigens	Peracetic acid	Highly effective in vitro (8 µg/mL ^¶^) Requires higher concentrations under industrially relevant conditions (400–1200 µg/mL ^¶^)	MIC range: 2–8 ppm ^¶^		[151,152,153,154]
		Reduction but no complete elimination of viable *Campylobacter* counts during poultry processing (40 or 100 µg/mL ^¶^ for 2 or 15 min)	^†^		[156]
		Tolerance observed in biofilms at a concentration of 8000 µg/mL ^¶^ for 23% of the poultry strains tested		Biofilm-mediated protection	[50]
		Completely inactivated biofilms (200 µg/mL ^¶^ for 45 s), but with efficacy dependent on concentration and exposure time and influenced by the presence of biofilm-associated microflora			[162]
Peroxygen compound mixture (organic peroxides)	Peracetic acid with peroxy-octanoic acid	No complete inactivation of biofilms (50 µg/mL ^¶^ for 180 s), with efficacy dependent on concentration and exposure time and influenced by the presence of biofilm-associated microflora	^†^	Biofilm-mediated protection	[162]
Alkaline agent/inorganic salt	Trisodium phosphate		MIC range: 2–16 µg/mL	-	[135]
		Significant reduction but no complete eradication from raw poultry at 120,000 µg/mL ^¶^ for 15 s of contact time	^†^		[157]
Anionic surfactant	Sodium dodecyl sulphate	-	MIC range: 128–1024 µg/mL ^§^	-	[135]

Susceptibility definition: ^a^ Empirical MIC distribution and previously cutoffs in related Gram-negative bacteria; ^b^ MIC distributions and comparative analyses with other bacterial organisms; ^c^ reduction rate of viable cells after 5 min of disinfectant contact > 10^5^ cells; ^d^ comparison of MIC values to those from positive control *C. jejuni* NCTC 11168 and detection of resistance genes. Implications for AMR: ^#^ >75% were resistant to both antibiotics and disinfectants tested; ^§^ higher tolerance in erythromycin-sensitive and non-multidrug-resistant strains. ^¶^ ppm, mg/L and % were converted to µg/mL assuming aqueous solutions for harmonization (1 ppm = 1 mg/L = 1 µg/mL; 1% = 10,000 µg/mL). ^†^ When MIC values were not reported, the metric type was classified as contact-time survival, defined as reduction or elimination following disinfectant exposure under specified time and concentration conditions.

## 5. Adaptive Responses and Resistance Mechanisms to Biocides in *Campylobacter* spp.

The impact of biocide exposure on the emergence of resistance and cross-resistance is difficult to predict, largely because experimental conditions often do not reflect real-world use, particularly regarding biocide concentration, time of exposure, or the presence of organic matter [63]. Accordingly, the selective pressures exerted by biocides in food production and processing environments may differ substantially from those observed under laboratory conditions. Even so, growing evidence suggests that exposure to biocides can contribute to AMR through different mechanisms [16]. Despite these concerns, stepwise exposure experiments complement the evidence obtained in real-world settings, supporting the concept that poultry-associated isolates, the most ecologically relevant reservoir of biocide-tolerant *Campylobacter*, can develop decreased susceptibility under sustained chemical pressure, and is a way of obtaining insights into adaptive responses and resistance mechanisms.

Biocides may promote resistance through several mechanisms, including (i) natural selection of tolerant or resistant strains; (ii) induction of adaptive phenotypes, both commonly associated with membrane alterations or changes in efflux pump expression; (iii) horizontal gene transfer, potentially enhanced by cell lysis and the release of DNA; and (iv) improved biofilm formation, which provides physical protection but can also act as a reservoir for resistance genes [16]. In *Campylobacter* spp., the implications of biocide exposure remain less explored, and key questions persist regarding the molecular and phenotypic bases of reduced susceptibility.

### 5.1. Adaptative Responses and Cross-Resistance Following Biocide Exposure

Stepwise exposure of *C. jejuni* and *C. coli* to increasing sub-inhibitory concentrations of five biocides (triclosan, benzalkonium chloride, cetylpyridinium chloride, chlorhexidine diacetate and trisodium phosphate) has been shown to induce partial tolerance not only to the selecting biocide but also, in some cases, to other biocides and antibiotics [48]. Both increases and decreases in MICs have been reported, highlighting the complexity and strain-dependent nature of adaptation. For instance, cross-resistance to erythromycin was observed in three *C. jejuni* strains, with MICs increasing up to eightfold after five passages with all the biocides, particularly with a significant increase for triclosan, benzalkonium chloride, and cetylpyridinium chloride [48]. However, in most tested combinations, no cross-resistance was detected, and in some cases, biocide-adapted strains exhibited increased susceptibility to antibiotics, an effect more evident in *C. coli* [48]. Active efflux mechanisms appear to contribute variably to biocide susceptibility, depending on the compound’s mode of action. Adapted strains also displayed changes in outer membrane protein profiles and cell morphology, supporting a multifactorial adaptation process [48]. Similarly, Techaruvichit et al. (2016) [49] demonstrated that stepwise adaptation of *C. jejuni* to trisodium phosphate, acetic acid, sodium hypochlorite, and two commercial acidic and alkaline biocides used in poultry processing led to modest but consistent increases in biocide MICs (1.1–1.7-fold). Importantly, this adaptation was accompanied by reduced susceptibility to antibiotics, particularly aminoglycosides, suggesting potential cross-resistance effects [49].

The type of biocide plays a key role in determining whether adaptive responses or irreversible cellular damage may occur. Chlorous acid has been shown to be approximately ten times more effective than sodium hypochlorite against *C. jejuni* and *C. coli*. Importantly, it maintains activity in chicken juice and induces bacterial death through protein damage [160]. In contrast, *C. jejuni* can recover following exposure to 64 ppm chlorine [155], whereas recovery after exposure to acidified sodium chlorite has not been observed [161]. A transcriptomic analysis provided mechanistic insight into these differences. Acidified sodium chlorite inhibited essential cellular functions, with marked disruption of membrane integrity, suppression of metabolic and respiratory pathways, impairment of antioxidant defenses, and activation of DNA and RNA damage responses, ultimately leading to cell death [161]. Conversely, chlorine exposure mainly altered membrane permeability, inducing oxidative stress and increased energy demands, with upregulation of genes involved in the TCA cycle, cellular respiration, and oxidoreductase activity, alongside a downregulation of ribosome biogenesis and cell growth, division and replication, particularly at 5 °C, thus inducing responses consistent with an adaptive survival strategy, particularly at low temperatures [161].

### 5.2. Molecular Determinants of Reduced Susceptibility

The mechanisms of reduced susceptibility to biocides are not widely understood, and relatively few biocide resistance genes have been identified, with most studies showing that these genes are often components of efflux pumps or potential efflux pump regulators [163]. In *Campylobacter*, the CmeABC and CmeDEF efflux systems are among the most extensively characterized mechanisms contributing to decreased susceptibility to biocides and antibiotics [48,164,165]. The involvement of these systems has been validated using efflux pump inhibitors and gene inactivation studies, which demonstrated reduced MICs for triclosan and benzalkonium chloride, particularly in strains with higher MICs. In turn, smaller effects were observed for chlorhexidine diacetate, cetylpyridinium chloride, and trisodium phosphate, indicating compound-specific mechanisms [48,165]. Exposure to acidified sodium chlorite has also been associated with increased expression of *cj1174* gene, encoding a membrane transporter of cations and cationic drugs, further implicating transport systems in biocide stress responses [161]. Nonetheless, efflux activity alone does not fully explain reduced susceptibility, as modifications in membrane structure and permeability also appear to play important roles [165].

Among disinfectant-associated resistance genes, QAC efflux pump genes such as *qacF*, *qacG*, *qacE* and *qacEΔ1*, which may be found in Gram-negative bacteria (found in plasmids), were searched [163,166]. Nonetheless, results have been conflicting with no clear association of the presence of the gene with the decreased susceptibility to biocides, such as benzalkonium chloride [163,166]. Although *qacEΔ1* has been detected in *C. jejuni* and *C. coli*, no clear correlation with increased tolerance to QACs, including benzalkonium chloride or cetrimonium bromide, has been demonstrated [134]. Importantly, the expression levels of these genes and the contribution of different allelic variants to reduced susceptibility in *Campylobacter* remain unexplored.

### 5.3. Persistence and Biofilm-Mediated Tolerance to Biocides

In *Campylobacter* species, oxidative stress is considered a major trigger for environmental persistence, by concurrently stimulating biofilm formation, but also inducing the transition of cells into the VBNC state, two closely interconnected survival strategies [167].

Biofilm formation represents an important phenotypic adaptation that enhances bacterial survival under adverse environmental conditions. *Campylobacter jejuni* is capable of forming biofilms on a variety of surfaces along the poultry production chain, with biofilm formation being strain-dependent and significantly enhanced in nutrient-rich environments such as chicken juice [50,159,168,169]. Besides the protective barrier that limits the penetration of biocides and antimicrobial agents, the proximity of bacterial cells has been shown to further enhance horizontal gene transfer, accelerating the spread of AMR [170,171]. Bacterial cells have also been reported to transition into the VBNC state, particularly under stress conditions such as refrigeration or nutrient limitation. In this state, cells may remain metabolically active and potentially virulent while evading routine culture-based detection [172,173]. The interplay between biofilm formation and the VBNC state may therefore increase bacterial tolerance to standard cleaning and disinfection procedures, enabling prolonged persistence on abiotic surfaces and contributing to the underestimation of contamination levels throughout the food chain [6,174,175]. Nevertheless, the role of these phenotypic adaptations in protecting *Campylobacter* against the effects of biocides remains insufficiently understood. This is particularly relevant since the physiological state of bacterial cells and their association with biofilms can influence their susceptibility to biocides used in food chain environments.

While environmental stress is frequently associated with the induction of the VBNC state in *Campylobacter*, the role of biocides in triggering this response remains largely understudied. However, available evidence indicates that chlorous acid does not promote VBNC formation in *C. jejuni* and *C. coli* [160]. In turn, the response of VBNC Campylobacter cells to exposure to biocides remains largely unknown.

Regarding biofilms, although sessile cells can be inactivated by biocides, their efficacy depends on the compound type, concentration, exposure time, temperature, and organic load, among others [50,158,162,176].

When evaluating the effects of hypochlorite, chlorhexidine, and peracetic acid on *C. jejuni* biofilm formation, the detection of strains with reduced susceptibility suggests that repeated or insufficient exposure to these agents may select for adaptive responses [50]. In that study, nearly one third of the analyzed strains (9/30) exhibited tolerance to at least one disinfectant routinely used in the poultry sector, namely peracetic acid (0.8%), sodium hypochlorite (1%), or chlorhexidine (1%) [50]. In contrast, chlorine was shown to achieve complete inactivation of preformed *C. jejuni* biofilms at relatively low concentrations and short contact times, whereas QACs, peroxyacetic acid, and mixtures of peroxyacetic acid with peroxy-octanoic acid acted more slowly and were less effective overall [162].

Sanitizer efficacy was further reduced by the presence of biofilm-associated microflora, such as *Pseudomonas* spp., or by mixed-species biofilms composed of different *C. jejuni* and/or *C. coli* strains, highlighting the importance of microbial interactions in modulating disinfectant performance [162,176]. Notably, benzalkonium chloride concentrations as high as 10% (*w*/*v*) were unable to completely inhibit biofilm formation or remove established biofilms after 10 min of exposure across temperatures ranging from 1 to 25 °C [158]. Moreover, the reduction in *C. jejuni* cells in benzalkonium chloride-treated preformed biofilms was strain-dependent, with surface water isolates displaying greater resistance than slaughterhouse-derived isolates [159]. Interestingly, biofilm-associated cells were reported to be more susceptible to benzalkonium chloride and exhibited lower efflux pump activity compared with planktonic cells, highlighting the complexity of biofilm-associated tolerance mechanisms [159]. Altogether, these results reinforce that the effectiveness of biocides against *C. jejuni* biofilms is highly dependent on the context, such as nutrient-rich conditions, but there is also strain dependence.

Biofilm formation therefore represents a key phenotypic tolerance mechanism, as the biofilm resistance to disinfection can be seen as a multifactorial event, where matrix can hinder disinfectant diffusion, reduce oxidative activity, and enable the persistence of metabolically less active bacterial cells, ultimately compromising sanitation outcomes [177,178]. In addition, enhancement of biofilm formation may contribute to phenotypic resistance to antibiotics [16]. The adaptation of *C. jejuni* to sublethal concentrations of biocides has been shown to induce marked variability in biofilm architecture, structure, and adhesion properties in the presence and absence of biocides, when compared with non-adapted cells [49]. While non-adapted cells formed sparse and small biofilm clusters, biocide-adapted cells often exhibited pronounced structural changes, including larger aggregates, rough or protuberant surfaces, and distinctive ice-crystal-like formations, particularly in acetic acid-adapted cells exposed to sodium hypochlorite or the acidic commercial biocide. A quantitative analysis revealed that biocide adaptation could either enhance or reduce biofilm formation, with increases in the biovolume of biofilm being more frequent than decreases, especially following exposure of sodium hypochlorite-adapted cells to biocides. Surface coverage and roughness also varied significantly depending on the adaptation and exposure combination, although no consistent patterns were observed. When changes in biofilm adhesion strength occurred, they were predominantly characterized by increased adhesion, suggesting enhanced surface attachment and persistence. Overall, these findings demonstrate that the adaptation to biocides influences biofilm formation [49]. Thus, beyond providing direct protection from biocides, repeated biocide exposure may promote adaptive changes that enhance biofilm-forming capacity and ultimately contribute to decreased susceptibility to other antimicrobial agents.

**Figure 1:**
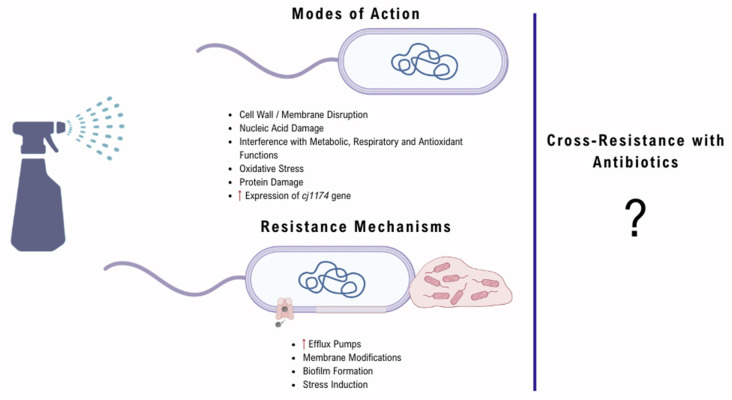
Proposed modes of action of biocides and potential bacterial resistance mechanisms in *Campylobacter* spp. The figure summarizes how biocide-induced cellular damage and bacterial adaptive responses may intersect with pathways involved in antibiotic resistance, emphasizing the need for further investigation into the potential link between biocide exposure and antimicrobial cross-resistance.

Taken together, the available evidence demonstrates that reduced susceptibility of *Campylobacter* spp. to biocides may arise from a combination of molecular mechanisms, including efflux, membrane modification or stress induction, as well as from phenotypic resistance like biofilms or ecological and environmental factors such as organic load or biocide-driven selection (Figure 1). These mechanisms often overlap with AMR mechanisms underscoring the need to evaluate biocide use as a potential driver of AMR in food production systems and in *Campylobacter* species specifically.

## 6. Biocide Residues, Environmental Monitoring, and Sanitation Strategies for Controlling *Campylobacter* in the Food Chain

Biocides employed for disinfection of food- and feed-contact surfaces may persist as residues capable of transferring to food products, potentially exposing bacterial populations to sublethal concentrations [179]. Such exposure may contribute to the persistence of *Campylobacter* in food-processing environments by promoting stress adaptation, transient tolerance mechanisms, or biofilm-associated survival. Addressing these risks requires integrated strategies that link regulatory monitoring with practical food-processing interventions.

Within the European Union, monitoring programs have been established to ensure that biocide residues in food remain within safe and acceptable limits [62]. However, surveillance data have detected QACs, including benzalkonium chloride and didecyldimethylammonium chloride, above recommended thresholds in certain food categories [83]. In contrast, a significant monitoring gap exists for other widely used biocides, such as peracetic acid and chlorine-based compounds, which may be applied in poultry chilling and washing procedures but lack harmonized reporting requirements [6,39,180]. Moreover, discrepancies between official Competent Authority data and Food Business Operator monitoring, where official controls frequently report higher contamination levels, further underscore the need for more rigorous oversight and standardized monitoring approaches [4,17].

Environmental monitoring is therefore essential to identify niches where *Campylobacter* may persist despite routine sanitation procedures [6,170,180]. These locations can act as reservoirs for strains capable of persisting under repeated exposure to cleaning and disinfection agents.

Effective control of foodborne pathogens, namely *Campylobacter*, requires coordinated interventions throughout the food chain, beginning at the primary production level. In this context, appropriate cleaning and disinfection protocols should be combined with preventive measures, such as vaccination programs, to reduce pathogen transmission and cross-contamination. Risk-based environmental monitoring programs are particularly important for validating hygiene zoning, assessing sanitation efficacy, and identifying potential contamination reservoirs. Emerging molecular tools, including next-generation sequencing and whole-genome sequencing, may offer valuable opportunities to improve the detection and tracking of contamination niches across the food chain [4,17,181].

From an operational perspective, both regulators and food processors should prioritize sanitation strategies that minimize the risk of tolerance development. Disinfection protocols should be validated under realistic processing conditions, particularly in the presence of high organic loads, that may reduce the efficacy of commonly used biocides and promote biofilm formation [6,39,170].

The adoption of combined or “hurdle-based” interventions may further enhance pathogen control, for example by pairing physical treatments such as ultrasound or steam with chemical disinfectants, or by combining modified atmosphere packaging with natural antimicrobial compounds [29,39,180]. Additionally, rotating classes of antimicrobials and implementing targeted biofilm-control strategies may help limit the emergence of tolerant populations and improve the removal of persistent contamination [6,39,170,182].

Despite the widespread use of biocides in food production systems, data on the occurrence and persistence of biocide residues relevant to *Campylobacter* control remain limited. Strengthened monitoring programs and targeted research are therefore needed to clarify the role of disinfectant exposure in the selection and persistence of tolerant *Campylobacter* populations within the food chain.

## 7. Conclusions

This review highlights the complex relationship between biocide exposure and the persistence of *Campylobacter* in food production environments. Although chemical disinfection remains a cornerstone of food safety, its improper or suboptimal application may unintentionally promote bacterial persistence in food production environments.

Evidence indicates that exposure to biocides can trigger adaptive responses in *Campylobacter* that may enhance survival under biocidal stress. These mechanisms contribute to functional tolerance that may compromise sanitation efficacy and potentially overlap with AMR pathways, thereby raising concerns about the possible role of biocides in the persistence and dissemination of resistant strains along the food chain.

Despite increasing evidence of such adaptive responses, important knowledge gaps remain. Current understanding of the extent to which biocides contribute to AMR co-selection, persistence, or dissemination is still limited. In particular, data are scarce on the effects of repeated and long-term exposure to biocides under realistic food-processing conditions, as well as the roles of stress-induced gene regulation, stable genetic changes, mobile genetic elements, microbial community interactions, and biofilm-associated survival in the shaping of AMR dynamics in *Campylobacter*.

Addressing these knowledge gaps will require coordinated research efforts. Future studies should prioritize the development of standardized methodologies for evaluating biocide susceptibility in *Campylobacter* under conditions that closely mimic industrial environments. The integration of phenotypic approaches with whole-genome sequencing and other genomic tools will be particularly valuable for identifying adaptive traits and understanding their evolutionary dynamics.

From a regulatory and public health perspective, strengthened surveillance frameworks and monitoring programs for biocide use and residues across the food production chain are also needed. Expanding monitoring to include widely used biocides, while aligning regulatory thresholds with genomic surveillance data, could improve the detection and management of adaptive and resistant *Campylobacter* strains.

Ultimately, bridging these knowledge gaps will be essential to better understand the role of biocides in shaping *Campylobacter* adaptation and to ensure the long-term effectiveness of antimicrobial interventions aimed at mitigating AMR and safeguarding food safety within a One Health framework.

## Data Availability

No new data were created or analyzed in this study. Data sharing is not applicable to this article.

## Abbreviations

The following abbreviations are used in this manuscript:

AMR	Antimicrobial Resistance
DMT	Drug/Metabolite Transporter
LPS	Lipopolysaccharide
MBC	Minimum Bactericidal Concentration
MFS	Major Facilitator Superfamily
MIC	Minimum Inhibitory Concentration
QACs	Quaternary Ammonium Compounds
RND	Resistance-Nodulation-Division
TCA	Tricarboxylic Acid
VBNC	Viable but Non-Culturable
WHO	World Health Organization
